# The [4Fe‐4S] clusters of Rpo3 are key determinants in the post Rpo3/Rpo11 heterodimer formation of RNA polymerase in *Methanosarcina acetivorans*

**DOI:** 10.1002/mbo3.399

**Published:** 2016-08-25

**Authors:** Matthew E. Jennings, Faith H. Lessner, Elizabeth A. Karr, Daniel J Lessner

**Affiliations:** ^1^ Department of Biological SciencesUniversity of ArkansasFayettevilleARUSA; ^2^ Department of Microbiology and Plant BiologyUniversity of OklahomaNormanOKUSA

**Keywords:** anaerobes, archaea, iron–sulfur cluster, transcription

## Abstract

Subunits Rpo3 and Rpb3/AC40 of RNA polymerase (RNAP) from many archaea and some eukaryotes, respectively, contain a ferredoxin‐like domain (FLD) predicted to bind one or two [4Fe‐4S] clusters postulated to play a role in regulating the assembly of RNAP. To test this hypothesis, the two [4Fe‐4S] cluster Rpo3 from *Methanosarcina acetivorans* was modified to generate variants that lack the FLD or each [4Fe‐4S] cluster. Viability of gene replacement mutants revealed that neither the FLD nor the ability of the FLD to bind either [4Fe‐4S] cluster is essential. Nevertheless, each mutant demonstrated impaired growth due to significantly lower RNAP activity when compared to wild type. Affinity purification of tagged Rpo3 variants from *M. acetivorans* strains revealed that neither the FLD nor each [4Fe‐4S] cluster is required for the formation of a Rpo3/11 heterodimer, the first step in the assembly of RNAP. However, the association of the Rpo3/11 heterodimer with catalytic subunits Rpo2′ and Rpo1″ was diminished by the removal of the FLD and each cluster, with the loss of cluster 1 having a more substantial effect than the loss of cluster 2. These results reveal that the FLD and [4Fe‐4S] clusters, particularly cluster 1, are key determinants in the post Rpo3/11 heterodimer assembly of RNAP in *M. acetivorans*.

## 1 Introduction

Iron–sulfur (Fe‐S) clusters are protein cofactors that serve diverse functions, including catalytic, sensing, structural, and electron transfer. The most common function of Fe‐S clusters is to serve as electron carriers during oxidation–reduction reactions catalyzed by metabolic enzymes, including those found in electron transport systems (Johnson, Dean, Smith, & Johnson, 2005). Fe‐S clusters are an ancient prosthetic group that likely served as the primary electron carriers in the anaerobes that were the dominant life form on Earth for approximately 2.5 billion years, when the atmosphere was largely devoid of oxygen (Imlay, 2006). The reliance of strict anaerobes on Fe‐S clusters is supported by genomic evidence, which has revealed that the genomes of extant strict anaerobes encode significantly more Fe‐S proteins, containing primarily [4Fe‐4S] clusters, than the genomes of extant aerobes (Major, Burd, & Whitman, 2004; Sousa et al., 2013). This disparity is likely due to the fact that Fe‐S clusters are typically oxygen labile. Nonetheless, Fe‐S clusters serve critical roles in the vast majority of anaerobes and aerobes.

Fe‐S clusters are also found in proteins that function in replication, transcription, and translation, processes where an electron carrier cofactor would not likely be necessary. The incorporation of Fe‐S clusters into proteins involved in the central dogma may provide mechanisms to correlate information processing systems with energy conserving processes requiring Fe‐S clusters within cells. For example, many of the enzymes that are involved in DNA replication in eukaryotes and archaea, including replicative polymerases, helicase, and primase, contain [4Fe‐4S] clusters (Fuss, Tsai, Ishida, & Tainer, 2015). Often the [4Fe‐4S] clusters serve as a structural determinant required for the activity of enzymes, such as in primase (Klinge, Hirst, Maman, Krude, & Pellegrini, 2007). The [4Fe‐4S] cluster is essential in the B‐family of DNA polymerases in yeast, where it is required for subunit interaction and polymerase complex stabilization (Netz et al., 2012). The crystal structure of RNA polymerase (RNAP) from the archaeon *Sulfolobus solfataricus* revealed a [4Fe‐4S] cluster coordinated by cysteine residues within domain 3 (D3) of Rpo3 (Hirata, Klein, & Murakami, 2008). Rpo3 is also referred to as RpoD or subunit D. However, Rpo followed by a number is the current classification for archaeal RNAP subunits (Werner, 2013). Many strictly anaerobic archaea possess a Rpo3 with a D3 containing two [4Fe‐4S] cluster‐binding motifs, which is homologous to 2[4Fe‐4S] cluster ferredoxin, a ubiquitous electron carrier protein (Rodriguez‐Monge, Ouzounis, & Kyrpides, 1998). Thus, D3 can also be referred to as the ferredoxin‐like domain (FLD). Rpo3 of RNAP from numerous archaea with diverse physiology contains a D3/FLD with one or two [4Fe‐4S] cluster‐binding motifs (Lessner, Jennings, Hirata, Duin, & Lessner, 2012). Moreover, the corresponding subunit in eukaryotic RNAPs (Rpb3/AC40) also contains D3 and several eukaryotes possess Rpb3/AC40 subunits with a D3/FLD predicted to bind a [4Fe‐4S] cluster (Hirata & Murakami, 2009; Hirata et al., 2008). The conservation of [4Fe‐4S] clusters in RNAPs from two domains of life across multiple taxa indicates that the clusters serve an important, but likely not essential, role in RNAP. The precise role the [4Fe‐4S] cluster(s) serve in RNAP is unknown.

RNAP is a conserved multisubunit complex found in all three domains of life (Ebright, 2000; Werner, 2008). Archaea and bacteria possess a single RNAP responsible for synthesizing all RNA, whereas eukaryotes possess between three to five types of RNAP that synthesize different subsets of RNA (Werner & Grohmann, 2011). Structurally, archaeal RNAP is most similar to eukaryotic RNAPII; each comprised of 12–13 subunits, including several subunits not present in bacterial RNAP, which is comprised of five subunits (Decker & Hinton, 2013; Jun, Reichlen, Tajiri, & Murakami, 2011). The α subunit of bacterial RNAP is homologous to Rpo3 and Rpb3/AC40 subunits of archaeal and eukaryotic RNAP, respectively. However, all α subunits from sequenced bacteria lack D3, comprising the FLD found in numerous Rpo3 and Rpb3/AC40 subunits (Hirata & Murakami, 2009). The structural similarity of archaeal and eukaryotic RNAP, along with the shared presence of D3/FLD, support a shared ancestry, one that incorporated the use of [4Fe‐4S] clusters in RNAP in certain lineages.

Rpo3 is located a substantial distance from the active site of archaeal RNAP, indicating that the [4Fe‐4S] cluster(s) likely do not directly participate in catalysis (Hirata et al., 2008; Korkhin et al., 2009). The [4Fe‐4S] cluster(s) more likely serve a structural or regulatory function. The homologous Rpo3/Rpb3/AC40/α subunits are each involved in the first step in the assembly of RNAP. In archaea, Rpo3 forms a heterodimer with Rpo11 (RpoL), which initiates the assembly of RNAP (Eloranta, Kato, Teng, & Weinzierl, 1998; Goede, Naji, von Kampen, Ilg, & Thomm, 2006). All other RNAP subunits sequentially assemble on the Rpo3/11 heterodimer. It was initially postulated that the [4Fe‐4S] cluster(s) are required for the ability of Rpo3 to from a heterodimer with Rpo11. This hypothesis was supported by recombinant studies with *S. solfataricus* RNAP. *Sulfolobus solfataricus* Rpo3 contains an oxygen‐stable [4Fe‐4S] cluster, and the removal of [4Fe‐4S] cluster binding impaired the ability to form a recombinant heterodimer with Rpo11 (Hirata et al., 2008). Unlike *S. solfataricus*, which is an aerobe, many strict anaerobes, such as methanogens, contain a D3/FLD predicted to bind two [4Fe‐4S] clusters. Previous studies with the methanogen *Methanosarcina acetivorans* demonstrated that Rpo3 is capable of binding two oxygen‐labile [4Fe‐4S] clusters that are not required for Rpo3/11 heterodimer formation, but the clusters affect the stability of the heterodimer, and thus may influence assembly of RNAP after the formation of the Rpo3/11 heterodimer (Lessner et al., 2012). In either case, the [4Fe‐4S] cluster(s) may serve as a structural determinant that is required for the optimal assembly of RNAP.

Herein, we have used the *M. acetivorans* genetic system to investigate the importance of the [4Fe‐4S] clusters to specific steps in the in vivo assembly of RNAP, and to delineate the significance of each cluster to RNAP assembly and activity. A combination of genetic and biochemical experiments revealed that neither the FLD nor the ability of the FLD to bind either [4Fe‐4S] cluster was required for Rpo3 to form a heterodimer with Rpo11, indicating the first step of RNAP assembly in *M. acetivorans* is not influenced by the FLD or the presence of the [4Fe‐4S] clusters. Moreover, the FLD and the ability to bind either [4Fe‐4S] cluster 1 or 2 are not essential to RNAP in *M. acetivorans*. However, our results demonstrate that the FLD and the ability to bind the [4Fe‐S] clusters are important for the interaction of the Rpo3/11 heterodimer with catalytic subunits of RNAP, and therefore, are important for the in vivo assembly of RNAP post Rpo3/11 heterodimer formation. In particular, the inability of Rpo3 to bind [4Fe‐4S] cluster 1 had a more substantial impact compared to the loss of [4Fe‐4S] cluster 2 on the assembly of RNAP in *M. acetivorans*. The greater importance of [4Fe‐4S] cluster 1 in Rpo3 from *M. acetivorans* provides support for the conservation of the analogous cluster in Rpo3/Rpb3/AC40 subunits from numerous archaea and eukaryotes, including *S. solfataricus* Rpo3.

## 2 Experimental Procedures

### 2.1 Classification of archaeal *rpo3* genes

The *M. acetivorans* Rpo3 protein sequence (P0CG28.1) was used in a BLASTP search of archaeal sequences in the nonredundant NCBI database. The returned sequences were screened and duplicates, or those not annotated as an RNAP subunit, were removed. The remaining sequences were aligned using the MEGA program (v 6.06) (Tamura, Stecher, Peterson, Filipski, & Kumar, 2013) to identify those sequences that contain a domain similar to domain 3, defined as aligning with residues 171–221 of the *M. acetivorans* sequence. Species were classified into six groups based on the presence of D3/FLD and arrangement of the cysteine residues comprising the predicted [4Fe‐4S] cluster‐binding motifs within the domain as described previously (Lessner et al., 2012).

### 2.2 Generation of Rpo3 variants and recombinant protein analyses

**Figure 1:**
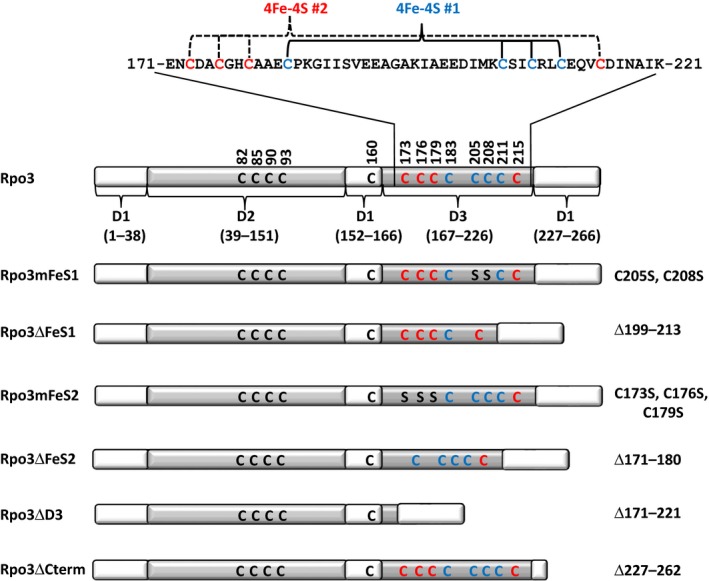
*Methanosarcina acetivorans* Rpo3 variants used in this study. Rpo3 is depicted to show the three domains (D1–3) and the sequence of D3/FLD, including the cysteines that comprise each [4Fe‐4S] cluster‐binding motif. Each variant is listed below the wild type to show which residues were changed (cysteine [C] to serine [S]) or deleted

A complete list of the plasmids and primers used in this study is included in Tables S1 and S2, respectively. The construction of *M. acetivorans rpo3* genes harboring mutations in domain 3 was done in pRpoDL, which is used for the coexpression of Rpo3 and C‐terminally His‐tagged Rpo11 in *Escherichia coli* (Lessner et al., 2012). Briefly, following the manufacturer's instructions, the QuikChange II Site‐Directed Mutagenesis Kit (Agilent Technologies) was used with pRpoDL as the template and primers were designed using the QuikChange Primer Design Program to generate variants of Rpo3 (Fig.**** 1) encoded in pRpoDL. Specifically, the *rpo3*ΔFeS1 and *rpo3*ΔFeS2 mutations were generated by deleting nucleotides 594–636 (cluster 1; primers QCRpoDΔFeS1For and QCRpoDΔFeS1Rev) and nucleotides 509–539 (cluster 2; primers QCRpoDΔFeS2For and QCRpoDΔFeS2Rev), respectively. Similarly, the *rpo3*mFeS1 and *rpo3*mFeS2 were generated by changing nucleotides 614 and 623 from G to C (cluster 1; primers QCRpoDmFeS1For and QCRpoDmFeS1Rev) and nucleotides 518, 527, and 536 from G to C (cluster 2; primers QCRpoDmFeS2For and QCRpoDmFeS2Rev), respectively. To generate the *rpo3*ΔCterm mutation, primers RpoDΔCtermFor and RpoDΔCtermRev were 5′ phosphorylated and used to amplify the entire pRpoDL plasmid minus nucleotides 679–783 of the *rpo3* sequence. The PCR product was then blunt ligated to generate pDL414. All of the constructs were confirmed by sequencing. Generation of pDL408 containing *rpo3* with the entire domain 3 deleted (*rpo3ΔD3*) has been described previously (Lessner et al., 2012).

Each Rpo3 variant was coexpressed with subunit L(His) in *E. coli* Rosetta (DE3) (pLacI) and Rpo3/11(His) heterodimers were purified anaerobically by Ni^2+^ affinity chromatography and size‐exclusion chromatography as described previously (Lessner et al., 2012). Fe‐S clusters were reconstituted into purified Rpo3/11(His) heterodimers as described previously (Cruz & Ferry, 2006).

### 2.3 Generation of merodiploid strains of *M. acetivorans* capable of inducible expression of (His)Rpo3

Primers were designed to amplify wild‐type and mutated *rpo3* genes from each respective derivative of pRpoDL (Table S2). The forward primer (HisRpoDNdeFor) contained an *Nde*I restriction site and an N‐terminal His‐tag, while the reverse primer (HisRpoDHindRev) contained a *Hind*III restriction site. PCR products were digested with *Nde*I and *Hind*III and ligated with similarly digested pJK027A (Guss, Rother, Zhang, Kulkarni, & Metcalf, 2008). For the *rpo3*ΔCterm construct, an alternative reverse primer was required (RpoDΔCtermHindRev). All of the derivatives of pJK027A were confirmed by DNA sequencing. Each derivative of pJK027A was used to transform *M. acetivorans* strain WWM73 as described previously (Metcalf, Zhang, Apolinario, Sowers, & Wolfe, 1997). The successful integration of the plasmid into the chromosome of the parent strain was confirmed by PCR (Guss et al., 2008). Each strain is capable of the tetracycline‐dependent expression of a separate (His)Rpo3 variant.

### 2.4 Generation of *M. acetivorans* mutants with *rpo3* replaced with *rpo3* encoding a (His)Rpo3 variant

The generation of mutant strains of *M. acetivorans*, where native *rpo3* is replaced with *rpo3* encoding a (His)Rpo3 variant, was attempted using homologous recombination with plasmids derived from pJK301 (Guss et al., 2008). Given the conflicting *Nde*I restriction enzyme site in the pJK301 sequence and the sequence surrounding *rpoD* in the chromosome, the QuikChange mutagenesis kit was used to remove the *Nde*I site from pJK301 (CATATG to CACATG) to generate pDL517. Similarly, 4 kb of *M. acetivorans* genomic DNA, which included the *rpo3* gene and the upstream 3 kb sequence, was cloned into pUC19 to create pDL518. The upstream region contains two natural restriction sites which would interfere with downstream cloning, both of which occur in genes encoding 30S ribosomal proteins (MA1109 and MA110). The QuikChange mutagenesis kit was used to introduce silent mutations into pDL518 which removed the restriction site from both genes but did not alter the encoded amino acid, resulting in the generation of pDL520. Primers QCRpoDUSXhoF and QCRpoDUSXhoR were used to remove the *Xho*I site from MA1109 (902 bp upstream of *rpo3* start site; CTCGAG to CTAGAG silent mutation for leucine codon) and primers QCRpoDUSApaF and QCRpoDUSApaR were used to remove the *Apa*I site from MA1110 (192 bp upstream of the *rpo3* start site; GGGCCC to GGACCC silent mutation for glycine codon).

Primers (RpoDUSApaFor and RpoDUSXhoRev) were used to amplify 2.8 kb of DNA encompassing *rpo3* at the 3′ end and approximately 2 kb of upstream sequence at the 5′ end, from pDL520. The primers also added *Apa*I and *Xho*I at the 5′ and 3′ ends, respectively, to the PCR product. The PCR product was digested with *Apa*I and *Xho*I and ligated with similarly digested pDL517 to create pDL521. Similarly, the 2.5 kb sequence downstream and adjacent to *rpo3* was amplified from genomic DNA with primers (RpoDDSBamFor and RpoDDSNotRev) that add a *Bam*HI and *Not*I restriction enzyme site at the 5′ and 3′ ends, respectively. The PCR product was digested with *Bam*HI and *Not*I and ligated with similarly digested pDL521 to generate pDL522. Plasmid pDL522 contains *rpoD* along with 2 kb upstream sequence and 2.5 kb sequence downstream of *rpo3*. Between the upstream and downstream regions is 2.1 kb of plasmid DNA including *pac*, which encodes resistance to puromycin. Thus, pDL522 and derivatives can be used to replace native *rpo3* with *rpo3* encoded in pDL522 by homologous recombination and selection with puromycin.

Derivatives of pDL522 encoding (His)Rpo3 variants were generated by amplifying each mutant *rpo3* from each pJK027A‐based plasmid (described above) using the primers (HisRpoDNdeFor and RpoDUSXhoRev), which added *Nde*I and *Xho*I restriction enzyme sites at the 5′ and 3′ ends of the PCR product, respectively. The PCR products were digested with *Nde*I and *Xho*I and ligated with similarly digested pDL522 to create the derivatives of pDL522, each containing *rpo3* encoding a separate (His)Rpo3 variant. All plasmids were verified by DNA sequencing.

*Methanosarcina acetivorans* strain WWM73 was transformed with pDL522 or a derivative as described previously (Metcalf et al., 1997). Multiple transformations were attempted with plasmids encoding (His)*rpo3* variants, and in each case a transformation utilizing pJK027A and pDL522 were included as positive controls for transformation efficiency and homologous recombination, to confirm that the lack of any observed transformants was not due to poor transformation and/or homologous recombination. Mutants were initially screened and identified by PCR, and each mutant confirmed by PCR amplification and sequencing of the entire *rpo3* gene.

### 2.5 Expression and purification of (His)Rpo3 variants in *M. acetivorans*

All of the following procedures were completed under anaerobic conditions in an anaerobic chamber (Coy Laboratories) containing 95% N_2_ and 5% H_2_ at 25°C unless otherwise noted. *Methanosarcina acetivorans* cells were grown in a stoppered flask at 37°C in 1 L of high‐salt (HS) medium supplemented with methanol (125 mmol/L) and 0.025% sodium sulfide (Sowers, Boone, & Gunsalus, 1993). For merodiploid strains, cultures were induced with 100 μg/ml tetracycline immediately upon inoculation. Cells were harvested at an optical density at 600 nm of 0.6–0.9 as described previously (Sowers, Baron, & Ferry, 1984). Pelleted cells were harvested and resuspended in lysis buffer (10 mmol/L Tris, 15% glycerol, 10 μmol/L ZnCl_2_, 2 mol/L KCl, 30 mmol/L MgCl_2_). Phenylmethylsulfonyl fluoride and benzamidine were added to the resuspended cells to a final concentration of 1 mmol/L. Cell pellets were stored under nitrogen in sealed vials at −80°C until use.

For the purification of (His)Rpo3, frozen cells were thawed on ice and lysed by sonication or by repetitive freeze/thaw cycles. DNase I was added to a final concentration of 4 μg/ml, and the lysate was incubated for 1 hr, followed by centrifugation for 15 min at 16,000*g*. The soluble fraction was filtered (0.45 μm pore size) and loaded onto a pre‐equilibrated (lysis buffer plus 10 mmol/L imidazole) Ni^2+^‐agarose resin column (600 μl). The flow‐through from the column was collected and reapplied to the column a total of four times. The column was then washed with 12 ml buffer (10 mmol/L Tris, 15% glycerol, 10 μmol/L ZnCl_2_, 0.1 mol/L KCl, 10 mmol/L imidazole), and bound protein was eluted from the column with the addition of 3 ml of elution buffer (20 mmol/L Tris, 15% glycerol, 10 μmol/L ZnCl_2_, 30 mmol/L MgCl_2_, 250 mmol/L imidazole). The eluates were aliquoted into sealed vials and stored under nitrogen at −80°C or used immediately.

### 2.6 Nonspecific transcription assays

Cell lysates and eluates were assayed for nonspecific transcription activity using a radiolabeled assay similar to those described previously (China & Nagaraja, 2010; Darcy et al., 1999; Kuhn, Bohne, Liere, Weihe, & Borner, 2007; Naji, Bertero, Spitalny, Cramer, & Thomm, 2008), but used a 90‐bp oligonucleotide as a template. Due to the observed loss of activity after freezing, all lysates and eluates were assayed prior to freezing. For lysate samples, *M. acetivorans* cells were grown and processed to obtain the soluble fraction as described earlier, except that the cells were resuspended in elution buffer. A transcription assay mixture was freshly prepared by adding 2 μl ^32^P‐UTP (20 μCi) and 2 μl 0.5 mol/L DTT (1 mmol/L) for every ml of transcription buffer (20 mmol/L Tris, 20 mmol/L MgCl_2_ 1 mmol/L ATP, 1 mmol/L GTP, 1 mmol/L CTP, 100 μg/ml 90 bp oligonucleotide, pH 8.0) as needed. The oligonucleotide sequence was randomly generated using a random oligonucleotide generator with the GC content set to 22%. The sequence used is shown in Table S2. Two complimentary oligonucleotides were synthesized (IDT) and dissolved to a concentration of 10 μg/μL in buffer (10 mmol/L Tris, 50 mmol/L NaCl, 1 mmol/L EDTA, pH 7.5), and annealed by combining equal amounts of each and incubating at 95°C for 5 min and then cooled to 25°C. Ten microliters of sample were mixed with the transcription assay mixture to a final volume of 100 μl and incubated at 35°C for 30 min. The reaction mixture was then spotted onto cellulose filter disks (23 mm) and washed in succession with 0.5 mol/L Na_2_HPO_4_ (7X) and water (3X). Filters were rinsed with 95% ethanol and air‐dried for 10 min before being placed in scintillation vials containing 10 ml scintillation fluid (National Diagnostic Ecoscint H). The vials were vortexed for 10 s, and radioactivity counts were measured for 1 min (CPM) on a Packard 1600 TR Liquid Scintillation Analyzer.

### 2.7 Analytical methods

Growth studies were performed with *M. acetivorans* strains in HS medium supplemented with 125 mmol/L methanol and 0.025% sodium sulfide as described previously (Sowers et al., 1993). Generation times were calculated from at least three replicate cultures. For recombinant proteins, concentrations were determined using the Bradford method (Bradford, 1976) and bovine serum albumin as a standard. The concentration of protein in imidazole eluates from (His)Rpo3 purifications was determined using the Invitrogen Qubit 2.0 Fluorometer and the Qubit Protein Assay Kit as directed by the manufacturer. The iron and acid‐labile sulfide content of Rpo3/11(His) heterodimers were determined as described previously (Cruz & Ferry, 2006). SDS‐PAGE and western blotting were performed by standard procedures, using anti‐Rpo3/11 antibody described previously (Lessner et al., 2012). Antibodies used to specifically detect Rpo3, subunit B′, or subunit A″ were supplied by Genscript and produced using synthesized peptides specific for each protein: Rpo3 (CISSDPKIQPADPNV), Rpo2′ (CGKTSPPRFLEEPSD), and Rpo1″ (CDGEVKQIGRHGISG). The intensity of bands in western blots was calculated using ImageJ (Schneider, Rasband, & Eliceiri, 2012). A standard curve to calculate the Rpo3 concentration in samples using western blot band intensity was generated using purified recombinant Rpo3.

## 3 Results

### 3.1 Conservation of [4Fe‐4S] cluster‐binding motifs in Rpo3 of archaeal RNAP

Previously, Rpo3 subunits (99) from the available sequenced archaeal genomes at the time were analyzed for the presence of D3/FLD and [4Fe‐4S] cluster motifs, which revealed six distinct groups (Lessner et al., 2012). Since this initial analysis, there are substantially more archaeal genome sequences available in the NCBI database, including sequences from recently identified phyla and orders. Thus, we have updated the Rpo3 classification and diversity table to include an additional 180 archaeal genome sequences (Table S3). A summary of the presence of D3/FLD and [4Fe‐4S] cluster motifs in Rpo3 among phyla/orders is shown in Table 1. Importantly, D3/FLD is present in Rpo3 from the majority of sequenced archaea, with the exceptions being all orders of the phylum Thaumarchaeota and all species in the order Methanococcales (Table 1). Of those archaea that have a D3/FLD‐containing Rpo3, the majority contain at least one [4Fe‐4S] cluster motif (12 of the 20 phyla/orders, Table 1). In particular, the cluster 1 motif is conserved, found in Rpo3 from 11 of the 12 phyla/orders. The cluster 2 motif is less conserved, typically only found in Rpo3 that also contain the cluster 1 motif. The cluster 1 motif, but not the cluster 2 motif, is found in the D3/FLD of the Rpb3/AC40 subunit from several eukaryotes (Hirata & Murakami, 2009; Hirata et al., 2008). Rpo3 with a FLD containing two complete [4Fe‐4S] binding motifs are found only in strictly anaerobic members of the Crenarchaeota and Euryarchaeota, as well as the recently deposited sequence from the founding member of the Lokiarchaeota (Spang et al., 2015). Since the D3/FLD and [4Fe‐4S] clusters are not universal among archaea, it is unlikely they are essential for the function of RNAP, but instead may play an accessory role. The higher prevalence of the cluster 1 motif among archaea, and its presence in the Rpb3/AC40 subunit of RNAP from several eukaryotes, indicates cluster 1 plays a more prominent role than cluster 2 in RNAP.

**Table 1:** Summary of the conservation of D3/FLD in Rpo3 from archaea

Phylum/Order	D3/FLD?	[4Fe‐4S] cluster‐binding motif	
		Cluster #1	Cluster #2
Crenarchaeota			
Desulfurococcales^b^	Yes	X^a^	X
Sulfolobales	Yes	X	
Thermoproteales	Yes		
Acidilobales^c^	Yes		
Fervidicoccales^c^	Yes	X	
Euryarchaeota			
Thermoplasmatales	Yes		
Archaeoglobales^c^	Yes	X	X
Halobacteriales	Yes		
Methanomassiliicoccales^c^	Yes	X	
Methanosarcinales^c^	Yes	X	X
Methanobacteriales^c^^*,*^ d	Yes	X	X
Methanococcales^c^	No		
Methanocellales^c^	Yes	X	X
Methanomicrobiales^c^	Yes	X	X
Methanopyrales^c^	Yes		
Natrialbales	Yes		
Thermococcales^c^	Yes		
Thaumarchaeota			
Nitrosopumilales	No		
Nitrososphaerales	No		
Cenarchaeales	No		
Nanoarchaeota^c^	Yes		
Korarchaeota	Yes		X
Parvarchaeota	Yes	X	
Lokiarchaeota	Yes	X	X

FLD, ferredoxin‐like domain.

a An X designates at least 60% of sequences belonging to species within the order possess the binding motif.

b Species classified as strict anaerobes contain both cluster motifs.

c All species are classified as strict anaerobes.

d Rpo3 in all sequenced Methanobacteriales contains cluster 1, and 56% also contain cluster 2.

### 3.2 Generation of Rpo3 variants deficient in binding [4Fe‐4S] cluster(s)

Previously, expression of Rpo3 deleted of D3/FLD, named Rpo3ΔD3 (previously DΔD3), in *E. coli* and within *M. acetivorans*, revealed that the FLD is not required for Rpo3 to form a heterodimer with Rpo11 (Lessner et al., 2012). To ascertain the importance of each [4Fe‐4S] cluster to Rpo3/11 heterodimer formation and the interaction of the Rpo3/11 heterodimer with other RNAP subunits during assembly of RNAP, Rpo3 variants were generated defective in binding cluster 1 or cluster 2. To specifically assess the effect of the absence of the cluster, but retention of the cluster‐binding region on Rpo3 interactions, cysteine residues predicted to coordinate the cluster were changed to serine residues, in addition to deletion mutants (Fig.**** 1). For example, Rpo3mFeS1 and Rpo3ΔFeS1 are each predicted to be incapable of binding cluster 1, but Rpo3mFeS1 still contains the cluster 1 binding region. As a control for subsequent in vivo studies, a Rpo3 variant deleted of C‐terminal residues 227–262 (Rpo3ΔCterm) was generated. These residues form an α‐helix required for the interaction of Rpo3 with Rpo11 (Hirata et al., 2008); thus, Rpo3ΔCterm should be unable to form a heterodimer with Rpo11.

First, to determine if each variant Rpo3 was capable of forming a heterodimer with Rpo11, each variant was coexpressed along with histidine‐tagged Rpo11 [Rpo11(His)] in *E. coli* followed by purification of Rpo11(His) using Ni^2+^ affinity and size‐exclusion chromatography. Similar to previous results obtained for the purification of recombinant Rpo3/11(His) and Rpo3ΔD3/11(His) heterodimers (Lessner et al., 2012), each single cluster‐binding Rpo3 variant was capable of forming a recombinant heterodimer with Rpo11(His) that was devoid of Fe‐S clusters (Fig. S1). However, coexpression of Rpo3ΔCterm with Rpo11(His) did not generate a heterodimer, but instead produced inclusion bodies containing Rpo3ΔCterm (data not shown), similar to results obtained when Rpo3 was expressed in the absence of Rpo11(His) (Lessner et al., 2012). These results are consistent with the C‐terminal helix of Rpo3 being required for association with Rpo3 and formation of the Rpo3/11 heterodimer, whereas mutation or deletion of the cluster 1 or cluster 2 binding regions does not impact formation of the Rpo3/11 heterodimer.

Next, to ascertain whether each variant Rpo3/11(His) heterodimer is competent in binding the predicted number of [4Fe‐4S] clusters, each purified variant Rpo3/11(His) heterodimer was reconstituted with iron and sulfide as previously described for Rpo3/11(His) and Rpo3ΔD3/11(His) (Lessner et al., 2012). Each Rpo3/11(His) heterodimer comprised a single cluster‐binding Rpo3 variant contained approximately four molecules of iron and sulfur after reconstitution (Table 2) and generated UV–visible spectra (Fig. S1) consistent with the presence of a single [4Fe‐4S] cluster. The ability to purify recombinant Rpo3/11(His) heterodimers competent in binding a single [4Fe‐4S] cluster indicates that the directed changes to the cluster 1 or 2 binding regions of Rpo3 specifically impact [4Fe‐4S] cluster incorporation. These results support the use of the single cluster‐binding Rpo3 variants, along with Rpo3ΔD3, to investigate the effect of the loss of cluster 1, 2, or the entire FLD on the in vivo assembly and activity of RNAP.

**Table 2:** Iron and sulfide content of purified recombinant Rpo3/11(His) heterodimers

Rpo3/11 heterodimer	Iron^a^	Sulfide^b^
Rpo3/11(His)^c^	8.3 ± 0.2	7.7 ± 0.8
Rpo3ΔD3/11(His)^c^	BDL	BDL
Rpo3ΔFeS1/11(His)	4.6 ± 0.1	3.3 ± 0.2
Rpo3mFeS1/11(His)	4.7 ± 0.2	3.5 ± 0.7
Rpo3ΔFeS2/11(His)	3.8 ± 0.1	3.0 ± 0.1
Rpo3mFeS2/11(His)	4.9 ± 0.2	3.3 ± 0.2

a nmol of iron/nmol of Rpo3/11(His) heterodimer.

b nmol of sulfide/nmol of Rpo3/11(His) heterodimer.

c Results from reference Lessner et al. (2012).

### 3.3 Loss of [4Fe‐4S] cluster binding in Rpo3 affects the in vivo assembly of RNAP

Previously, two merodiploid strains (DJL30 and DJL31) of *M. acetivorans* were generated (Table 3), each capable of tetracycline‐inducible expression of a second Rpo3 harboring an N‐terminal histidine tag to facilitate purification by Ni^2+^ affinity chromatography. Purification of (His)Rpo3ΔD3 from strain DJL31 showed that the FLD of Rpo3 is not required for the in vivo interaction with Rpo11 and subsequent formation of the Rpo3/11 heterodimer (Lessner et al., 2012). To specifically test the effect of the absence of cluster 1 or 2 within Rpo3 on the in vivo assembly of RNAP, four additional merodiploid strains of *M. acetivorans* were generated, each capable of expressing a (His)Rpo3 deficient in [4Fe‐4FS] cluster binding (Table 3). A fifth strain (DJL40) capable of expressing (His)Rpo3ΔCterm was also generated to use as a negative control because Rpo3ΔCterm cannot form a heterodimer with Rpo11. Similar to previous results obtained with strains DJL30 and DJL31 (Lessner et al., 2012), western blot analysis of induced and uninduced cells of strains DJL32–35 and DJL40 showed comparable levels of Rpo3 (data not shown), indicating expression of (His)Rpo3 does not significantly increase intracellular Rpo3 levels. Each strain exhibited wild‐type growth rates and cell yields when grown in the presence or absence of tetracycline (data not shown), indicating expression of each (His)Rpo3 variant is not detrimental to *M. acetivorans*.

**Table 3:** *Methanosarcina acetivorans* strains utilized in this study

Strain designation	Relevant genotype
DJL30^a^	*rpo3* merodiploid: contains tetracycline‐inducible (His)Rpo3
DJL31^a^	*rpo3* merodiploid: contains tetracycline‐inducible (His)Rpo3ΔD3
DJL32	*rpo3* merodiploid: contains tetracycline‐inducible (His)Rpo3ΔFeS1
DJL33	*rpo3* merodiploid: contains tetracycline‐inducible (His)Rpo3ΔFeS2
DJL34	*rpo3* merodiploid: contains tetracycline‐inducible (His)Rpo3mFeS2
DJL35	*rpo3* merodiploid: contains tetracycline‐inducible (His)Rpo3mFeS1
DJL40	*rpo3* merodiploid: contains tetracycline‐inducible (His)Rpo3ΔCterm
DJL51	Native *rpo3* replaced with *rpo3* encoding (His)Rpo3
DJL52	Native *rpo3* replaced with *rpo3* encoding (His)Rpo3ΔFeS1
DJL54	Native *rpo3* replaced with *rpo3* encoding (His)Rpo3ΔD3
DJL55	Native *rpo3* replaced with *rpo3* encoding (His)Rpo3mFeS2

a Previously generated (Lessner et al., 2012).

**Figure 2:**
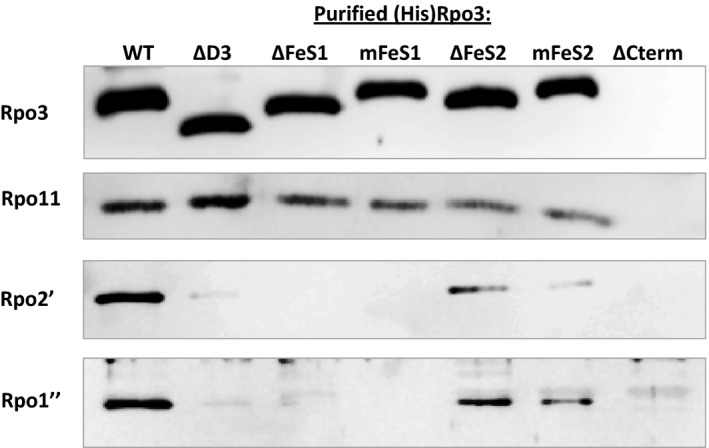
Analysis of the copurification of endogenous Rpo11, Rpo2′, and Rpo1″ with (His)Rpo3 variants expressed in *Methanosarcina acetivorans*. Separate SDS‐PAGE gels were loaded with samples of each imidazole eluate containing 15 ng of each (His)Rpo3. The gels were analyzed by western blot with antibodies specific for Rpo3, Rpo11, Rpo2′, or Rpo1″. For the imidazole eluate from the (His)Rpo3ΔCterm purification the gels were loaded with 1.5 μg of total protein since (His)Rpo3ΔCterm was not detected in the eluate

To test the ability of each (His)Rpo3 variant to compete with native Rpo3 for assembly into RNAP, each strain was grown under inducing conditions and each (His)Rpo3 variant purified from cell lysate by anaerobic Ni^2+^ affinity chromatography. The imidazole eluates obtained from the purification columns contained similar amounts of total protein, ranging from 50 to 180 ng μl^−1^. Western blot analysis using anti‐Rpo3 antibodies revealed the presence of (His)Rpo3 in all eluates, except the eluate generated from strain DJL40 expressing (His)Rpo3ΔCterm (Fig. 2). The lack of detection of (His)Rpo3ΔCterm was expected based on the inability of recombinant Rpo3ΔCterm to form a heterodimer with Rpo11(His) within *E. coli*. Thus, (His)Rpo3ΔCterm within *M. acetivorans* is likely subjected to proteolysis. Since the eluates contained contaminating protein as determined by Coomassie staining (data not shown), the concentration of (His)Rpo3 in each imidazole eluate was calculated using a calibration curve of band intensity with western blots containing known amounts of recombinant Rpo3. Multiple purifications revealed that the imidazole eluate containing (His)Rpo3 typically contained the highest amount of Rpo3, whereas the eluates containing (His)Rpo3 FLD variants contained 2‐20X less Rpo3. The concentration of Rpo3 in imidazole eluates from a representative experiment is shown in Table 4. These results indicate each [4Fe‐4S] cluster (His)Rpo3 variant is expressed in a stable form within *M. acetivorans*.

**Table 4:** Purification of (His)Rpo3 from merodiploid strains of *Methanosarcina acetivorans*

Strain/Rpo3	(His)Rpo3 (ng/μl) in eluate^a^	RNAP activity^b^
DJL30/(His)Rpo3	15.6 ± 1.7	2721 ± 413
DJL31/(His)Rpo3ΔD3	2.9 ± 0.4	BDL^c^
DJL32/ (His)Rpo3ΔFeS1	2.5 ± 0.5	BDL^c^
DJL35/(His)Rpo3mFeS1	1.0 ± 0.2	BDL^c^
DJL33/(His)Rpo3ΔFeS2	3.8 ± 1.2	BDL^c^
DJL34/(His)Rpo3mFeS2	0.6 ± 0.1	BDL^c^

a Mean ± SD of replicate samples.

b Activity (counts per min ng^−1^ Rpo3) determined using nonspecific transcription assay.

c Below detection limit of assay.

First, to determine if the inability to bind cluster 1 or 2 affects the in vivo formation of the Rpo3/11 heterodimer, SDS‐PAGE gels were loaded with eluate samples containing equal amounts of each (His)Rpo3 and examined for the presence of native Rpo11 by western blot. Although some variability in band intensity was observed, native Rpo11 was detected at a significant level in all of the normalized (His)Rpo3 samples (Fig. 2), revealing neither the presence of the FLD nor the ability to bind either cluster 1 or 2 is required for Rpo3 to interact with Rpo11 to form a stable heterodimer. Next, to ascertain whether the inability to bind cluster 1 or 2, or the loss of the entire FLD, affects the assembly of RNAP post Rpo3/11 heterodimer formation, samples normalized to (His)Rpo3 were examined by western blot using antibodies specific for Rpo2′ (RpoB′) or Rpo1″ (RpoA″) (Fig. 2). Rpo2′ and Rpo1″ comprise part of the catalytic core of RNAP (Hirata et al., 2008; Werner & Grohmann, 2011). Rpo2′ and Rpo1″ were detected in the eluates containing (His)Rpo3, (His)Rpo3ΔD3, (His)Rpo3ΔFeS2, and (His)Rpo3mFeS2, but differed significantly in the observed band intensity. Rpo2′ and Rpo1″ were barely detectable in the (His)Rpo3ΔD3 eluate, and (His)Rpo3ΔFeS2 and (His)Rpo3mFeS2 eluates contained approximately 50% less Rpo2′ and Rpo1″ than the (His)Rpo3 eluate, based on band intensity (Fig. 2). Rpo2′ and Rpo1″ were not detected in the eluates containing (His)Rpo3ΔFeS1 or (His)Rpo3mFeS1. These results reveal that the FLD domain is critical to the interaction of the Rpo3/11 heterodimer with at least Rpo2′ and Rpo1″ in *M. acetivorans*. In particular, the inability of (His)Rpo3ΔFeS1/11 or (His)Rpo3mFeS1/11 heterodimers to compete with native Rpo3/11 heterodimer for assembly with Rpo2′ and Rpo1″ reveals that the presence of cluster 1 is a key determinant for the assembly of RNAP after Rpo3/11 heterodimer formation in *M. acetivorans*. Finally, only the eluate containing (His)Rpo3 exhibited nonspecific transcription activity (Table 4), consistent with (His)Rpo3 being assembled into functional RNAP. The lack of transcription activity with the eluates containing (His)Rpo3ΔFeS1 and (His)Rpo3mFeS1 was expected as these eluates lack RNAP catalytic subunits Rpo2′ and Rpo1″ (Fig. 2). The lack of activity with imidazole eluates containing (His)Rpo3ΔD3, (His)Rpo3ΔFeS2, and (His)Rpo3mFeS2 could be due to the lack of fully assembled RNAP, assembled RNAP is largely inactive, and/or the level of fully assembled RNAP is insufficient to detect activity. Nonetheless, these results demonstrate that (His)Rpo3 is assembled into functional RNAP and reveal the importance of the FLD, in particular the cluster 1 region, of Rpo3 to the assembly of RNAP after the formation of Rpo3/11 heterodimer.

### 3.4 Neither the FLD nor the ability of the FLD to bind [4Fe‐4S] cluster 1 or 2 are essential to RNAP in *M. acetivorans*

The results from the purification of each (His)Rpo3 variant from the merodiploid strains of *M. acetivorans* suggest that the entire FLD region and the ability to bind cluster 1 are critical to the assembly of RNAP. To determine whether the FLD region and binding of the [4Fe‐4S] clusters are essential for the in vivo function of RNAP, we attempted to replace *rpo3* in the chromosome of *M. acetivorans* with mutated *rpo3* encoding each (His)Rpo3 variant. After several independent transformation experiments (*n* = 6), four mutant strains were obtained (Table 3). Native Rpo3 was replaced with (His)Rpo3, (His)Rpo3ΔD3, (His)Rpo3ΔFeS1, and (His)Rpo3mFeS2, revealing that neither [4Fe‐4S] cluster nor the entire FLD region are essential for functional RNAP in *M. acetivorans*.

**Figure 3:**
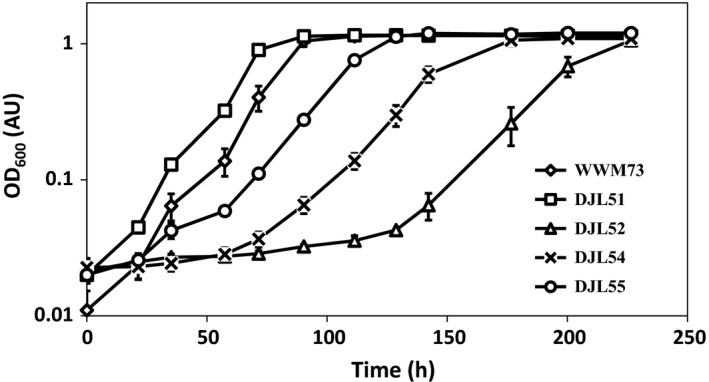
Comparison of the growth of *Methanosarcina acetivorans* mutants with methanol. Data points represent the mean ± standard deviation of triplicate cultures

The mutant strains were first analyzed by comparing their growth with methanol to that of the parent strain (WWM73) (Fig. 3, Table 5). Strain DJL51 grew identical to strain WWM73, indicating the addition of the histidine tag to the N‐terminus of Rpo3 does not affect RNAP assembly and activity. However, strains DJL52, DJL54, and DJL55 all grew slower than both WWM73 and DJL51, revealing that the FLD region and the ability to bind clusters 1 and 2 are required for the optimal assembly and/or activity of RNAP in *M. acetivorans*. In addition, strains DJL52 and DJL54 took significantly longer to reach the exponential phase of growth, with DJL52 typically having a lag phase six times longer in duration than strain DJL51 (Table 5). Despite the longer lag phase duration and slower generation times, strains DJL52, DJL54, and DJL55 each reached a similar final cell density (Table 5).

**Table 5:** Growth parameters of *Methanosarcina acetivorans* strains with methanol

Strain/Rpo3	Lag phase duration (hr)	Generation time (hr)	Maximum OD_600_
WWM73/Rpo3	≤20	9.9 ± 0.7	1.2 ± 0.03
DJL51/(His)Rpo3	≤20	9.4 ± 0.5	1.2 ± 0.05
DJL54/(His)Rpo3ΔD3	≥85	16.5 ± 0.3^a^	1.1 ± 0.1
DJL52/(His)Rpo3ΔFeS1	≥135	17.1 ± 0.4^a^	1.1 ± 0.02
DJL55/(His)Rpo3mFeS2	≥60	14.5 ± 0.2^a^	1.2 ± 0.03

Values are average ± standard deviation of triplicate cultures.

a Significant difference in generation time versus DJL51 lysate (*p* < .05).

### 3.5 Loss of the FLD or each [4Fe‐4S] cluster decreases the in vivo level of functional RNAP due to impaired assembly and/or stability of RNAP

**Figure 4:**
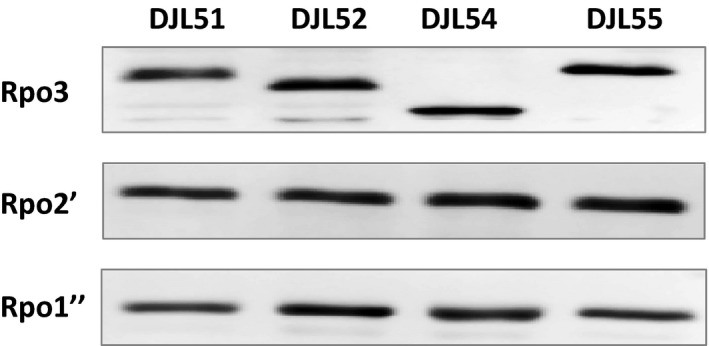
Comparison of the levels of RNAP subunits Rpo3, Rpo2′, and Rpo1″ in cell lysate from *Methanosarcina acetivorans* mutants by western blot. Separate SDS‐PAGE gels were loaded with samples containing 20 μg of total protein (Rpo3 blot), or normalized to contain 15 ng of Rpo3 (Rpo2′ and Rpo1″ blots)

**Figure 5:**
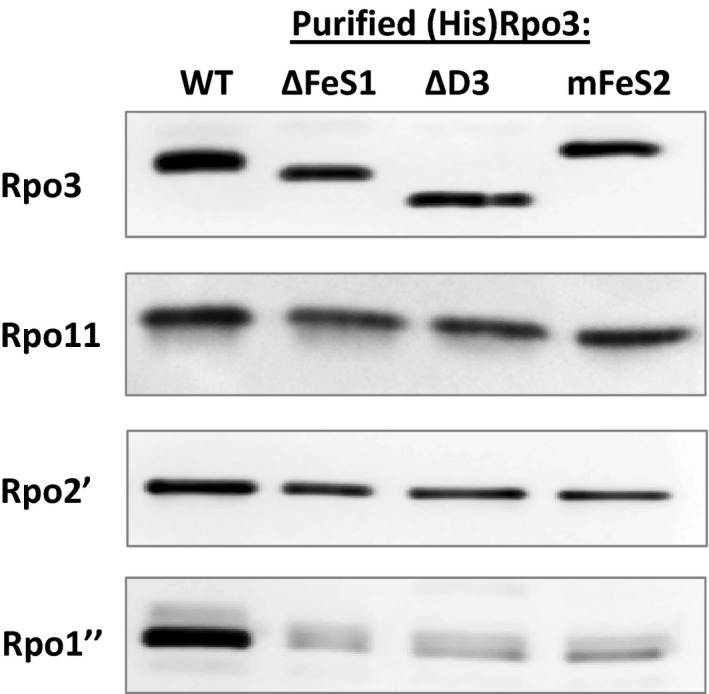
Analysis of the copurification of Rpo11, Rpo2′, and Rpo1″ with (His)Rpo3 variants purified from *Methanosarcina acetivorans* mutant strains. Separate SDS‐PAGE gels were loaded with samples of each imidazole eluate normalized to Rpo3; 15 ng (Rpo3 blot), 20 ng (Rpo11 blot), 10 ng (Rpo2′ blot), and 15 ng (Rpo1″ blot)

To determine if the assembly and/or activity of RNAP is affected by the mutations in the FLD regions of Rpo3, the levels of Rpo3, Rpo2′, and Rpo1″ in each mutant strain were compared. Cell‐free lysates derived from actively growing cultures of each strain contained similar levels of Rpo3, as determined by western blot of normalized total protein (Fig. 4). These results reveal that neither the loss of the entire FLD region nor the inability of Rpo3 to bind cluster 1 or 2 affects the in vivo amount of Rpo3. Next, to compare the levels of catalytic subunits Rpo2′ and Rpo1′′ in each strain, lysate samples containing an equal amount of Rpo3 (15 ng) for each strain were analyzed by western blot using anti‐Rpo2′ and anti‐Rpo1″ antibodies. There were no significant differences in the amounts of Rpo2′ and Rpo1′′ in lysates from multiple cultures (an example blot is shown in Fig. 4), indicating the in vivo levels of the RNAP catalytic subunits are not impacted by changes to the FLD of Rpo3. However, despite each strain containing similar levels of the individual RNAP subunits, lysate derived from strains DJL54, DJL52, and DJL55 exhibited significantly lower nonspecific transcription activity compared to strain DJL51, which contains a wild‐type FLD region (Table 6). In particular, lysates containing (His)Rpo3ΔD3 and (His)Rpo3ΔFeS1 contained the lowest activity, consistent with the results obtained from the purification of the (His)Rpo3 variants from the merodiploid strains (Fig. 2), which showed the greater importance of cluster 1 to the assembly of RNAP after the formation of the Rpo3/11 heterodimer. These results indicate that the assembly and/or the intrinsic activity of RNAP is negatively impacted by the removal of the FLD region or mutation of the cluster 1 or 2 binding sites of Rpo3. To specifically examine the impact on assembly, the (His)Rpo3 variant from strains DJL51, DJL52, DJL54, and DJL55 was purified using Ni^2+^ affinity chromatography. The levels of Rpo3, Rpo11, Rpo2′, and Rpo1″ in each imidazole eluate were determined by western blot (Fig. 5). Each eluate contained similar concentrations of (His)Rpo3 (Table 7). Western blot analysis of eluate samples normalized to contain the same amount of (His)Rpo3 revealed a similar level of Rpo11 in each sample (Fig. 5). Thus, the formation of the Rpo3/11 heterodimer is not altered by mutation of the FLD region of Rpo3 in the gene replacement strains, consistent with results obtained from the merodiploid strains (Fig. 2).

**Table 6:** RNAP activity in lysate from *Methanosarcina acetivorans rpo3* replacement strains

Strain/Rpo3	(His)Rpo3 (ng/μl) in lysate^a^	RNAP activity^b^
DJL51/(His)Rpo3	3.9 ± 0.1	8182 ± 165
DJL54/(His)Rpo3ΔD3	5.5 ± 0.5	2216 ± 128 (27%^c^)^d^
DJL52/ (His)Rpo3ΔFeS1	5.2 ± 2.3	2765 ± 253 (34%^c^)^d^
DJL55/(His)Rpo3mFeS2	6.5 ± 2.0	4505 ± 586 (55%^c^)^d^

a Mean ± SD of replicate samples.

b Activity (counts per min ng^−1^ Rpo3) determined using nonspecific transcription assay.

c Percent activity of DJL51 lysate.

d Significant difference in activity versus DJL51 lysate (*p* < .05).

**Table 7:** Purification (His)Rpo3 from *rpo3* replacement strains of *Methanosarcina acetivorans*

Strain/Rpo3	(His)Rpo3 (ng/μl) in eluate^a^	RNAP activity^b^
DJL51/(His)Rpo3	6.7 ± 0.6	210 ± 14
DJL54/(His)Rpo3ΔD3	6.6 ± 0.1	90 ± 20 (43%^b^)^d^
DJL52/(His)Rpo3ΔFeS1	9.8 ± 0.4	33 ± 9 (16%^c^)^d^
DJL55/(His)Rpo3mFeS2	5.5 ± 0.1	94 ± 10 (45%^c^)^d^

a Mean ± SD of replicate samples.

b Activity (counts per min ng^−1^ Rpo3) determined using nonspecific transcription assay.

c Percent activity of (His)Rpo3 sample.

d Significant difference in activity versus (His)Rpo3 sample (*p* < .05).

Surprisingly, unlike the partial purification of (His)Rpo3 variants from the merodiploid strains, which revealed heterodimers containing (His)Rpo3ΔD3 and (His)Rpo3ΔFeS1 were defective in assembling with Rpo2′ (Fig. 2), Rpo2′ copurified with (His)Rpo3ΔD3 and (His)Rpo3ΔFeS1 almost as equally as well as it did with (His)Rpo3 (Fig. 5). However, the copurification of Rpo1″ with (His)Rpo3ΔD3, (His)Rpo3ΔFeS1, and (His)Rpo3mFeS2 was significantly less than that observed with (His)Rpo3. Consistent with the diminished copurification of Rpo1″, the eluates containing (His)Rpo3ΔD3, (His)Rpo3ΔFeS1, and (His)Rpo3mFeS2 exhibited significantly lower RNAP activity compared to the eluate containing (His)Rpo3 (Table 7). These results indicate that (His)Rpo3ΔD3, (His)Rpo3ΔFeS1, and (His)Rpo3mFeS2 form at least a Rpo3/11/2′ subcomplex, but that the loss of the FLD or the inability to bind either cluster results in a subcomplex that is impaired in associating with at least Rpo1″.

## 4 Discussion

**Figure 6:**
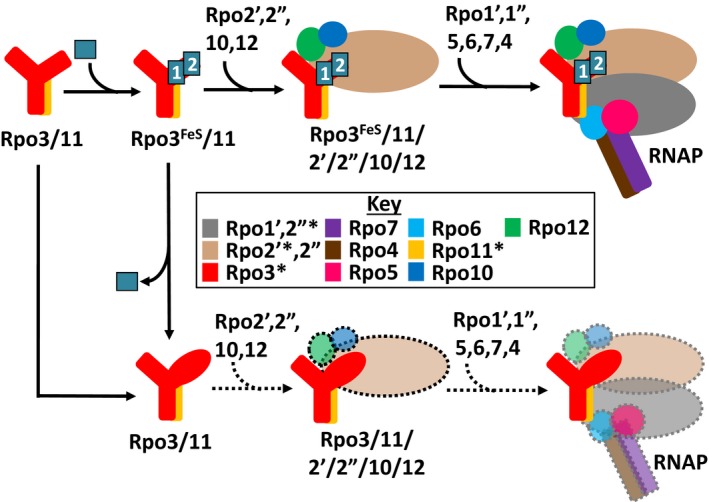
Model of the impact of the [4Fe‐4S] clusters in the FLD of Rpo3 on the formation of RNAP in *Methanosarcina acetivorans*. Assembly of the two [4Fe‐4S] clusters (boxes labeled 1 and 2) occurs after formation of the Rpo3/11 heterodimer [10]. The lack of cluster incorporation into or cluster loss from the Rpo3/11 heterodimer negatively impacts assembly and/or stability Rpo3/11/10/12/2′/2″ subcomplex. The absence of the cluster(s) alters the conformation of the Rpo3/11/10/12/2′/2″ subcomplex and impacts the association with Rpo1″, decreasing assembly and/or stability of complete RNAP. Decreased assembly and/or stability is indicated by the transparent subunits and dashed lines. The asterisks denote RNAP subunits specifically analyzed in this study

The results presented herein provide insights into the role [4Fe‐4S] clusters play in specific steps during the assembly of RNAP in *M. acetivorans*. The assembly of RNAP within cells from all three domains of life occurs in stages, starting with the formation of an assembly platform. The assembly of archaeal RNAP starts with the formation of the Rpo3/11 heterodimer, followed by the stepwise addition of the remaining subunits (Eloranta et al., 1998; Goede et al., 2006). The Rpo3/11 heterodimer first associates with Rpo10 (RpoN) and Rpo12 (RpoP), followed by the addition of catalytic core subunit Rpo2, to form an Rpo3/11/10/12/2 (DLNPB) subcomplex. Rpo2 is split into two separate proteins (Rpo2′ and Rpo2″) in many Euryarchaeota, including *M. acetivorans*. Thus, *M. acetivorans* specifically forms a Rpo3/11/10/12/2′/2″ subcomplex. The Rpo3/11/10/12/2 subcomplex is competent in transcription factor interaction and DNA binding, indicating it is highly stable (Goede et al., 2006). Rpo1′ and Rpo1″, which make up the other portion of the catalytic core, subsequently associate with the Rpo3/11/10/12/2 complex, followed by auxiliary subunits Rpo5 (RpoH), Rpo6 (RpoK), Rpo4 (RpoF), and Rpo7 (RpoE) to form complete RNAP. Given that the [4Fe‐4S] cluster(s) reside in Rpo3, which is not part of the catalytic core of RNAP, and that the [4Fe‐4S] cluster in the structures of archaeal RNAP is a substantial distance from the active site (Hirata et al., 2008; Korkhin et al., 2009), the cluster(s) likely do not participate in catalysis. A more likely function for the cluster(s) is to serve as a determinant to regulate the de novo assembly of RNAP. Alternatively, the cluster(s) may serve as a recognition element for the specific interaction of RNAP with general or gene‐specific transcription factors. The essentiality of the clusters and their importance to specific steps in the assembly of RNAP within *M. acetivorans* was specifically investigated and the results obtained support the model proposed in Figure**** 6.

The purification of (His)Rpo3 variants from merodiploid strains of *M. acetivorans* revealed that neither the removal of the entire FLD nor the deletion or mutation of each [4Fe‐4S] cluster‐binding motif impacts the ability of Rpo3 to form a heterodimer with Rpo11 within *M. acetivorans* (Fig. 2). These results, combined with the ability to incorporate the [4Fe‐4S] clusters in vitro into the recombinant Rpo3/11 heterodimers that purify lacking clusters (Table 2 and Lessner et al., 2012), indicate insertion of each cluster occurs after the formation of the Rpo3/11 heterodimer (Fig. 6). In contrast, recombinant *S. solfataricus* Rpo3 with mutations in the [4Fe‐4S] cluster‐binding motif failed to form a heterodimer with Rpo11 in *E. coli*, indicating the single cluster (analogous to cluster 1 in *M. acetivorans*) is required for Rpo3/11 heterodimer formation in *S. solfataricus* (Hirata et al., 2008). However, *S. solfataricus* is an extremophile and its Rpo3 contains a disulfide bond in place of a second [4Fe‐4S] cluster, unlike *M. acetivorans* (Hirata et al., 2008). Mutation of the [4Fe‐4S] cluster‐binding motif in *S. solfataricus* Rpo3 may alter the conformation of D3 impacting the formation of the disulfide bond in the reducing environment of the cytoplasm of *E. coli*. Moreover, Rpo3 from the methanogen *Methanobrevibacter smithii* contains an FLD predicted to bind only cluster 1, similar to *S. solfataricus* Rpo3, and coexpression studies in *E. coli* revealed that the presence of a [4Fe‐4S] cluster is not required for the association of recombinant *M. smithii* Rpo3 with Rpo11 (unpublished results, SC Granderson and DJ Lessner). Although the requirement of the cluster(s) for the formation of the Rpo3/11 heterodimer cannot be ruled out in some species, the clusters are not required for the association of Rpo3 and Rpo11 in two methanogens, and likely the majority of other species containing Rpo3 predicted to bind [4Fe‐4S] clusters.

Results from the purification of (His)Rpo3 variants from the merodiploid strains of *M. acetivorans* revealed that the removal of the entire FLD and the deletion or mutation of the [4Fe‐4S] cluster 1 binding motif severely impacts the ability of the Rpo3/11 heterodimer to associate with Rpo2′ (Fig. 2). Thus, the formation of the Rpo3/11/10/12/2′/2″ subcomplex is likely compromised within *M. acetivorans*. Since the FLD of Rpo3 is in close proximity to Rpo2 in the structures of archaeal RNAP (Hirata et al., 2008; Korkhin et al., 2009), this result is consistent with the FLD being needed for optimal interaction with Rpo2′ in *M. acetivorans*. Previous results demonstrated that oxidative loss of the clusters from the FLD resulted in substantial change to the conformational stability of the *M. acetivorans* Rpo3/11 heterodimer (Lessner et al., 2012). The absence of clusters, in particular cluster 1 shown here, likely alters the confirmation of the FLD which negatively impacts interaction with Rpo2′, and thus formation of the Rpo3/11/10/12/2′/2″ subcomplex within *M. acetivorans* (Fig. 6). However, the ability to replace native Rpo3 with (His)Rpo3 variants revealed that the FLD, including each cluster, is not an essential determinant for the interaction of the Rpo3/11 heterodimer with Rpo2′ and likely the formation of the Rpo3/11/10/12/2′/2″ subcomplex in *M. acetivorans*. Unlike in the merodiploid strains, where each (His)Rpo3 variant must compete with native Rpo3 for association with native Rpo2′, each (His)Rpo3 variant must associate with native Rpo2′, as well as all other RNAP subunits, in order to obtain a viable gene replacement strain. Purification of (His)Rpo3ΔD3, (His)DΔDFeS1, and (His)Rpo3mFeS2 from the gene replacement strains revealed each variant forms a Rpo3/11 heterodimer that is competent in associating with Rpo2′ to likely form a Rpo3/11/10/12/2′/2″ subcomplex similar to (His)Rpo3 (Fig. 5). However, all three strains harboring mutations in the FLD of Rpo3 exhibited impaired growth and diminished RNAP activity compared to the control strain, with the strain containing (His)DΔDFeS1 having the most severe phenotype (Fig. 3 and Table 6). The diminished copurification of Rpo1″ with all three (His)Rpo3 FLD variants (Fig.**** 5) revealed that the observed phenotypes were due, at least in part, to impaired assembly after Rpo3/11/10/12/2′/2″ subcomplex formation. Thus, although deletion or mutation of the FLD of Rpo3 allows formation of a Rpo3/11/10/12/2′/2″ subcomplex, the subcomplex is impaired in the ability to associate with Rpo1″, and likely Rpo1′. Even though Rpo3 does not directly interact with Rpo1″ (Hirata et al., 2008), alteration of the FLD region could have long‐range effects on the interaction of the subcomplex with Rpo1″, likely due to altered association of Rpo3 with Rpo2′, which does directly contact Rpo1″. It is also possible that RNAP stability is altered such that Rpo1″ is lost during the purification of each (His)Rpo3 FLD variant. In either case, these results reveal that the absence of the FLD and each [4Fe‐4S] cluster has a substantial effect on the association of the Rpo3/11/2′ (likely Rpo3/11/10/12/2′/2″) with subunit Rpo1″, and possibly the remaining subunits (Rpo1′, Rpo5, Rpo6, Rpo7, and Rpo4). However, since the presence of the additional RNAP subunits (Rpo10, Rpo12, Rpo2″, Rpo1′, Rpo5, Rpo6, Rpo7, and Rpo4) was not analyzed, it cannot be rule out that the observed effects are due to the absence or diminished assembly of these subunits. Nonetheless, the FLD and [4Fe‐4S] clusters appear critical to the post Rpo3/11/2′ (likely Rpo3/11/10/12/2′/2″) subcomplex formation steps in the assembly of RNAP in *M. acetivorans* (Fig.**** 6).

The results from this study clearly demonstrate that [4Fe‐4S] cluster 1 is a more important determinant than [4Fe‐4S] cluster 2 for optimal formation of RNAP in *M. acetivorans*. Rpo2′ and Rpo1″ copurified with (His)Rpo3ΔFeS2 and (His)Rpo3mFeS2 from the merodiploid strains, but failed to copurify with (His)Rpo3ΔFeS1 and (His)Rpo3mFeS1 (Fig.**** 2). Moreover, strain DJL52 encoding (His)Rpo3ΔFeS1 had the most impaired growth of any of the strains (Fig. 3 and Table 5). These results are consistent with the [4Fe‐4S] cluster 1 motif being more conserved, compared to [4Fe‐4S] cluster 2 motif, in Rpo3 among archaea, and may explain why the cluster 1 motif, but not the cluster 2 motif, is found in the Rpb3/AC40 subunit of RNAP in some eukaryotes. The FLD and clusters are clearly not essential, since they can be deleted from *M. acetivorans* and are not found in all RNAPs. This supports a regulatory role for the clusters, possibly in the assembly of RNAP. The acquisition of [4Fe‐4S] clusters to use as determinants in the assembly of RNAP may allow *M. acetivorans*, and other species, to correlate energy conservation processes (e.g., methanogenesis) that are dependent on iron and Fe‐S clusters with biosynthetic processes (e.g., transcription) by altering the levels of functional RNAP. The [4Fe‐4S] clusters may be used to sense changes in key environmental factors that affect energy conservation, such as iron availability, oxygen, and reactive oxygen species.

D3/FLD is absent from all species of the order Methanococcales and the phylum Thaumarchaeota, both of which are deeply rooted archaeal lineages (Brochier‐Armanet, Forterre, & Gribaldo, 2011). Since ferredoxin is a critical Fe‐S cluster protein in methanogenesis (Ferry, 1999; Thauer, Kaster, Seedorf, Buckel, & Hedderich, 2008), and to the metabolism of most anaerobes, we hypothesize that two [4Fe‐4S] cluster ferredoxin were spliced into RNAP in an ancestral anaerobe (e.g., methanogen). This hypothesis is supported by the observation that only archaea classified as strict anaerobes possess Rpo3 with two [4Fe‐4S] cluster‐binding motifs, similar to ferredoxin. The FLD was subsequently modified due to diverse selective pressures exerted upon specific lineages, resulting in cluster modification (e.g., oxygen stability) or loss of one or both clusters. Due to the greater importance of cluster 1 to *M. acetivorans* RNAP shown here, cluster 2 was lost in more lineages than cluster 1. Although the results reveal the [4Fe‐4S] cluster(s) are key determinants in the formation of RNAP, it remains unclear whether the [4Fe‐4S] cluster(s) also serve as a recognition element for the interaction of RNAP with general and specific transcription factors to specifically alter the expression of certain genes. Importantly, the generation of *M. acetivorans* strains harboring RNAP missing the FLD or each [4Fe‐4S] cluster provides an avenue to address the significance of the [4Fe‐4S] clusters to the transcription of specific genes in the future.

## Funding Information

This work was supported in part by grant number P30 GM103450 from the National Institute of General Medical Sciences of the National Institutes of Health (D. J. L.), NSF grant number MCB1121292 (D. J. L.), NASA Exobiology grant number NNX12AR60G (D. J. L.), and the Arkansas Biosciences Institute (D. J. L.), the major research component of the Arkansas Tobacco Settlement Proceeds Act of 2000.

## Conflict of Interest

None declared.
