# Supplementary material for: The [4Fe‐4S] clusters of Rpo3 are key determinants in the post Rpo3/Rpo11 heterodimer formation of RNA polymerase in Methanosarcina acetivorans
Source: Microbiologyopen. 2016 Aug 25;6(1):e00399. doi: 10.1002/mbo3.399 (PMC5300874; doi:10.1002/mbo3.399)
Supplement: Supplementary file 1 [file MBO3-6-0-s001.pdf]

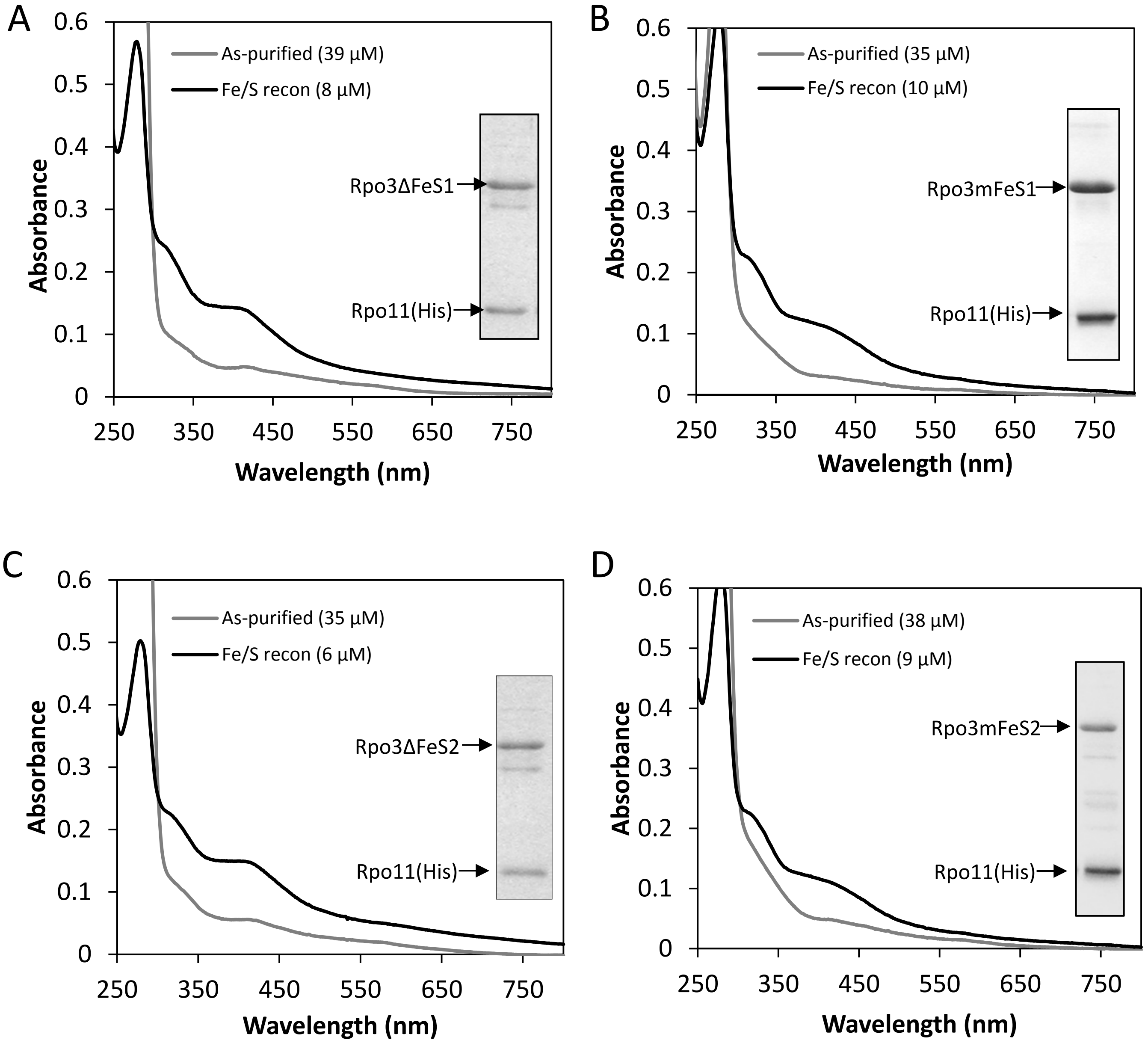

Fig. S1. UV-visible spectra and SDS-PAGE analysis of purified recombinant cluster-binding variant Rpo3/Rpo11(his) heterodimers. Each panel shows the UV-visible spectrum of the as-purified heterodimer and after reconstitution of the heterodimer with Fe and S. The inset shows the analysis of the purified heterodimer by 12% SDS-PAGE. A: Rpo3ΔFeS1/11(His); B: Rpo3mFeS1/11(His); C: Rpo3ΔFeS2/11(His); D: Rpo3mFeS2/11(His).
