# Supplementary material for: The [4Fe‐4S] clusters of Rpo3 are key determinants in the post Rpo3/Rpo11 heterodimer formation of RNA polymerase in Methanosarcina acetivorans
Source: Microbiologyopen. 2016 Aug 25;6(1):e00399. doi: 10.1002/mbo3.399 (PMC5300874; doi:10.1002/mbo3.399)
Supplement: Supplementary file 2 [file MBO3-6-0-s002.docx]

**Table S1: Plasmids utilized in this study**

| Plasmid | Description |
| --- | --- |
| pRpoDL | Plasmid containing *M. acetivorans rpoD* and *rpoL*(His) (Lessner *et al.*, 2012) |
| pDL408 | *rpoD*ΔD3 in pRpoDL backbone |
| pDL406 | *rpoD*ΔFeS1 in pRpoDL backbone |
| pDL407 | *rpoD*ΔFeS2 in pRpoDL backbone |
| pDL315 | *rpoD*mFeS1 in pRpoDL backbone |
| pDL314 | *rpoD*mFeS2 in pRpoDL backbone |
| pDL414 | *rpoD*ΔCterm in pRpoDL backbone |
| pJK027A | Plasmid for integration into *M. acetivorans* strain WWM73 chromosome (Guss *et al.*, 2008) |
| pDL516 | (His)*rpoD* cloned into pJK027A |
| pDL409 | (His)*rpoD*ΔD3 cloned into pJK027A |
| pDL415 | (His)*rpoD*ΔFeS1 cloned into pJK027A |
| pDL416 | (His)*rpoD*ΔFeS2 cloned into pJK027A |
| pDL418 | (His)*rpoD*mFeS1 cloned into pJK027A |
| pDL417 | (His)*rpoD*mFeS2 cloned into pJK027A |
| pDL420 | (His)*rpoD*ΔCterm cloned into pJK027A |
| pJK301 | Plasmid for double homolgous recombintation into *M. acetivorans* strain WWM73 chromosome (Guss *et al.*, 2008) |
| pDL517 | pJK301 derivative without NdeI site |
| pDL518 | pUC19 with 4 Kb of *M. acetivorans* genomic DNA (region upstream and including rpoD) cloned into BamHI site |
| pDL519 | pDL518 with genomic ApaI site upstream of *rpoD* removed by QuikChange |
| pDL520 | pDL519 with genomic XhoI site upstream of *rpoD* removed by QuikChange |
| pDL521 | pDL517 with 2.8 Kb of genomic DNA upstream of *rpoD* from pDL520 cloned into ApaI/XhoI sites |
| pDL522 | pDL521 with 2.8 Kb of *M. acetivorans* genomic DNA (region downstream of *rpoD*) cloned into BamH1/NotR sites |
| pDL525 | (His)*rpoD* cloned into pDL522 |
| pDL524 | (His)*rpoD*ΔD3 cloned into pDL522 |
| pDL523 | (His)*rpoD*ΔFeS1 cloned into pDL522 |
| pDL528 | (His)*rpoD*ΔFeS2 cloned into pDL522 |
| pDL527 | (His)*rpoD*mFeS1 cloned into pDL522 |
| pDL526 | (His)*rpoD*mFeS2 cloned into pDL522 |
|  |  |

**Table S2: Primers utilized in this study**

| Primer | Sequence | Description |
| --- | --- | --- |
| RpoDNdeFor | ggggaattaaggcatatgacgatggaag | forward primer to amplify *rpoD* with NdeI site at 5' end |
| RpoDHindRev | aagctcaaaagcttggcatagg | reverse primer to amplify *rpoD* with HindIII site at 3' end |
| HisRpoDNdeFor | attaaggcatatgcatcatcatcatcatcatacgatggaagtagacattct | forward primer to amplify *rpoD* with N-terminal His6 tag and NdeI site at 5' end |
| HisRpoDHindRev | ggtggtaagctttcagagctggtccagaattgc | reverse primer to amplify *rpoD* with HindIII site and stop codon at 3' end |
| QCRpoDΔFeS1For | gtggacttctatgaaaactcttttg | forward primer to make *rpoD*ΔFeS1 via QuikChange |
| QCRpoDΔFeS1Rev | ggcaatcttagctccggcctcttc | reverse primer to make *rpoD*ΔFeS1 via QuikChange |
| QCRpoDΔFeS2For | gaagaggccggagctaagattg | forward primer to make *rpoD*ΔFeS2 via QuikChange |
| QCRpoDΔFeS2Rev | aatggtaattacaggcatgtttttg | reverse primer to make *rpoD*ΔFeS2 via QuikChange |
| QCRpoDmFeS2For | accattgaaaactccgatgcctccggacactctgcggca | forward primer to make *rpoD*mFeS2 via QuikChange |
| QCRpoDmFeS2Rev | tgccgcagagtgtccggaggcatcggagttttcaatggt | reverse primer to make *rpoD*mFeS2 via QuikChange |
| QCRpoDmFeS1For | aagacatcatgaagtcttccatctccaggctctgtgagca | forward primer to make *rpoD*mFeS1via QuikChange |
| QCRpoDmFeS1Rev | tgctcacagagcctggagatggaa gacttcatgatgtctt | reverse primer to make *rpoD*mFeS1 via QuikChange |
| RpoDΔCtermFor | attctggaccagctctgaggatc | forward primer to amplify entire pRpoDL plasmid to generate *rpoD*ΔCterm |
| RpoDΔCtermRev | ttcatagaagtccactttgatcgcg | reverse primer to amplify entire pRpoDL plasmid to generate *rpoD*ΔCterm |
| RpoDΔCtermHindRev | ggtggtaagctttcagagctggtccagaatttc | reverse primer to amplify *rpoD*ΔCterm with HindIII site and stop codon at 3' end |
| C2Achr1 | gaagcttccccttgaccaat | Integration screening primer of ΦC31 site in *M. acetivorans* strain WWM73 (Guss *et al.*, 2008) |
| C2Achr1 | ttgattcggataccctgagc | Integration screening primer of ΦC31 site in *M. acetivorans* strain WWM73 (Guss *et al.*, 2008) |
| plscreen3 | gcaaagaaaagccagtatgga | Integration screening primer of ΦC31 site in *M. acetivorans* strain WWM73 (Guss *et al.*, 2008) |
| plscreen4 | tttttcgtctcagccaatcc | Integration screening primer of ΦC31 site in *M. acetivorans* strain WWM73 (Guss *et al.*, 2008) |
| QCJK301For | gaatctaaatggaggtttagacacatgcttgaaagactgaaagactc | Forward primer to remove NdeI site from pJK301 via QuikChange |
| QCJK301Rev | gagtctttcagtctttcaagcatgtgtctaaacctccatttagattc | Reverse primer to remove NdeI site from pJK301 via QuikChange |
| RpoDUSNcoFor | gatgatccatggatgttgaaccgcccttttctg | forward primer to amplify 4 Kb genomic region upstream of *rpoD* with NcoI site at 5' end |
| RpoDUSNcoRev | ggtggtccatggagaattctctgaataattcgc | reverse primer to amplify 4 Kb genomic region upstream of *rpoD* with NcoI site at 3' end |
| RpoDUSBamFor | ggcggcggatccatcatcgactgcggcatatctcccgc | forward primer to amplify 2.8 Kb genomic region upstream of *rpoD* with BamHI site at 5' end |
| RpoDUSBamRev | agccggatcctcagagctggtccagaattgccagc | reverse primer to amplify 2.8 Kb genomic region upstream of *rpoD* with BamHI site at 3' end |
| QCRpoDUSApaF | gagaagtcccggacccggtgcacag | forward primer to remove ApaI site from genomic DNA via QuikChange |
| QCRpoDUSApaR | ctgtgcaccgggtccgggacttctc | reverse primer to remove ApaI site from genomic DNA via QuikChange |
| QCRpoDUSXhoF | caggaaggaggactagagggccactacag | forward primer to remove XhoI site from genomic DNA via QuikChange |
| QCRpodUSXhoR | ctgtagtggccctctagtcctccttcctg | reverse primer to remove XhoI site from genomic DNA via QuikChange |
| RpoDUSApaFor | gctgctgggcccagggcagatgttgaaccgccttttc | forward primer to amplify 2.8 Kb upstream genomic region with ApaI at 5' end |
| RpoDUSXhoRev | agccctcgagtcagagctggtccagaattgccagc | reverse primer to amplify 2.8 Kb upstream genomic region with XhoI at 5' end |
| RpoDDSBamFor | ggcggattctgtcttcttattttgagaactcttaagg | forward primer to amplify ~2 Kb genomic region downstream of *rpoD* with BamHI at 5' end |
| RpoDDSNotRev | cgacgacgagcggccgcgcccaccctcactgtggagccggaacc | reverse primer to amplify ~2 Kb genomic region downstream of *rpoD* with NotI at 5' end |
| TxnAsy90A | atcttaatagttattatttctataaccttt ttaagtatccggtggtggatatctttc ataaatgaaaatatttttcgttgataattataa | Oligonucleotide for non-specific transcription assay |
| TxnAsy90B | ttataattatcaacgaaaaatattttcatttatgaaagatatccaccaccggatacttaaaaaggttatagaaataataactattaagat | Complimentary oligonucleotide for non-specific transcription assay |
|  |  |  |
